# Using dipstick urinalysis to predict development of acute kidney injury in patients with COVID-19

**DOI:** 10.1186/s12882-022-02677-y

**Published:** 2022-02-01

**Authors:** Meredith C. McAdams, Michael Li, Pin Xu, L. Parker Gregg, Jiten Patel, Duwayne L. Willett, Ferdinand Velasco, Christoph U. Lehmann, S. Susan Hedayati

**Affiliations:** 1 Division of Nephrology, Department of Medicine, University of Texas Southwestern Medical Center, 5939 Harry Hines Blvd, MC 8516, Dallas, TX 75390 USA; 2 University of Texas Southwestern College of Medicine, Dallas, TX USA; 3 Section of Nephrology, Department of Medicine, Selzman Institute for Kidney Health, Baylor College of Medicine, Houston, TX USA; 4 Section of Nephrology, Michael E. DeBakey Veterans Affairs Medical Center, Houston, TX USA; 5 Development Center for Innovations in Quality, Effectiveness, and Safety, Veterans Affairs Health Services Research, Houston, TX USA; 6 Parkland Hospital and Health System, Dallas, TX USA; 7 Division of Cardiology, Department of Medicine, University of Texas Southwestern Medical Center, Dallas, TX USA; 8 Texas Health Resources, Dallas, TX USA; 9 Clinical Informatics Center, University of Texas Southwestern Medical Center, Dallas, TX USA

**Keywords:** AKI, COVID-19, Proteinuria, Hematuria, Urinalysis, Predictive model

## Abstract

**Background:**

Acute kidney injury **(**AKI) is a common complication in patients hospitalized with COVID-19 and may require renal replacement therapy (RRT). Dipstick urinalysis is frequently obtained, but data regarding the prognostic value of hematuria and proteinuria for kidney outcomes is scarce.

**Methods:**

Patients with positive severe acute respiratory syndrome-coronavirus 2 (SARS-CoV2) PCR, who had a urinalysis obtained on admission to one of 20 hospitals, were included. Nested models with degree of hematuria and proteinuria were used to predict AKI and RRT during admission. Presence of Chronic Kidney Disease (CKD) and baseline serum creatinine were added to test improvement in model fit.

**Results:**

Of 5,980 individuals, 829 (13.9%) developed an AKI during admission, and 149 (18.0%) of those with AKI received RRT. Proteinuria and hematuria degrees significantly increased with AKI severity (*P* < 0.001 for both). Any degree of proteinuria and hematuria was associated with an increased risk of AKI and RRT. In predictive models for AKI, presence of CKD improved the area under the curve (AUC) (95% confidence interval) to 0.73 (0.71, 0.75), *P* < 0.001, and adding baseline creatinine improved the AUC to 0.85 (0.83, 0.86), *P* < 0.001, when compared to the base model AUC using only proteinuria and hematuria, AUC = 0.64 (0.62, 0.67). In RRT models, CKD status improved the AUC to 0.78 (0.75, 0.82), *P* < 0.001, and baseline creatinine improved the AUC to 0.84 (0.80, 0.88), *P* < 0.001, compared to the base model, AUC = 0.72 (0.68, 0.76). There was no significant improvement in model discrimination when both CKD and baseline serum creatinine were included.

**Conclusions:**

Proteinuria and hematuria values on dipstick urinalysis can be utilized to predict AKI and RRT in hospitalized patients with COVID-19. We derived formulas using these two readily available values to help prognosticate kidney outcomes in these patients. Furthermore, the incorporation of CKD or baseline creatinine increases the accuracy of these formulas.

**Supplementary Information:**

The online version contains supplementary material available at 10.1186/s12882-022-02677-y.

## Introduction

Coronavirus disease 2019 (COVID-19) has spread rapidly across the globe, causing a world-wide pandemic associated with high morbidity and mortality in affected patients [1]. Although primarily affecting the lungs, COVID-19 may also injure other organ systems [2]. Kidney involvement is common and may range from isolated proteinuria to severe acute kidney injury (AKI) [3, 4]. While the exact mechanism by which the virus induces kidney damage remains unclear, several processes have been postulated including direct viral invasion of tubular epithelia, podocyte damage, cytokine storm, and complement activation [5]. AKI is a major risk factor for mortality in patients hospitalized with COVID-19 [6].

Simple predictive models that use readily available kidney laboratory parameters, such as urinary dipstick results and serum creatinine, are lacking. Such models would not only allow clinicians to easily prognosticate the development of AKI and the need for renal replacement therapy (RRT) but may also provide insight into the pathophysiology of AKI associated with COVID-19.

Although several studies reported proteinuria and hematuria in conjunction with COVID-19 infection [7–16], only a few evaluated differences in the incidence of proteinuria and hematuria on dipstick urinalysis in patients with AKI as compared to those without AKI [9–11, 13, 17]. Even fewer studies investigated the association between proteinuria and hematuria with development of AKI and need for RRT [8]. One study reported that a urine protein-to-creatinine ratio (UPCR) ≥ 1 g/g on admission was associated with an increased risk of requiring RRT; albeit, these results may not be readily generalizable as UPCR is not one of the commonly obtained laboratory tests at admission [18]. Most of the other studies had small sample sizes, ranging from 129–307 total participants [8, 16, 19, 20], and two did not have a non-AKI control group [12, 21]. A larger, recently published study reported that presence of proteinuria and hematuria ≥ 1 + was associated with an increased risk of AKI [17] but did not exclude AKI present on admission, limiting the ability to predict development of AKI during hospitalization with COVID-19.

Given these knowledge gaps, the aims of our study were to [1] validate, in a large, racially and ethnically diverse cohort of patients with COVID-19, the association of increasing levels of dipstick proteinuria and hematuria with the development of AKI during hospitalization; [2] develop predictive models and formulas for AKI and RRT using clinically readily available dipstick proteinuria and hematuria results obtained upon hospitalization; [3] investigate whether the addition of baseline serum creatinine and/or Chronic Kidney Disease (CKD) presence to proteinuria and hematuria improved the prognostication of AKI and RRT in COVID-19 inpatients.

## Methods

### Study Design and Participants

We conducted a longitudinal study using the University of Texas Southwestern (UTSW) *COVID-19 Registry Collaborative* database which contains demographic information and clinical and laboratory data for all inpatient admissions for COVID-19 at UTSW Clements University Hospital, Parkland Hospital, and 18 hospitals in the Texas Health Resources Health System. The protocol was approved by the Institutional Review Board at UTSW as exempted human research. Informed consent was not required. We included all individuals who were 18 years of age or older, had a positive SARS-CoV2 PCR, were hospitalized between 3/1/2020 and 1/1/2021 with a COVID-associated billing diagnosis, and had admission urinalysis and serum creatinine results. We used the first hospitalization after the first positive SARS-CoV2 PCR or up to 10 days prior for analysis. If an individual had multiple admissions, only the first hospitalization was used. We excluded patients with a diagnosis of End Stage Kidney Disease (ESKD) or kidney transplantation.

### Clinical and laboratory variables

We extracted demographic information, past medical history, and laboratory variables from the Electronic Health Record (EHR) systems (EPIC systems, Verona, WI) including data pertaining to RRT during hospitalization. For patients with multiple urinalyses, we included only the initial urinalysis. As patients were admitted at 20 different hospitals, urinalysis data contained varying references ranges. Therefore, we recoded values to include urinalysis blood cutoffs as negative, trace, small, moderate, and large; and urinalysis protein cutoffs of negative, trace, 30 mg/dL, 100 mg/dL, and ≥ 300 mg/dL. Values of negative and trace were collapsed as “negative” for both hematuria and proteinuria variables. If a different numerical value was reported for proteinuria, the value was rounded down to the corresponding cutoff; 10 mg/dL and 20 mg/dL were included in negative category, 50 mg/dL and 70 mg/dL in the 30 mg/dL category, and 200 mg/dL in the 100 mg/dL category. Baseline creatinine was determined using a predetermined algorithm adapted and modified from a published algorithm [21–23], based on availability or absence of prior serum creatinine values (Additional Fig. 1**).** We categorized patients as having pre-existing CKD if the diagnosis was listed in the EHR record.

**Fig. 1:**
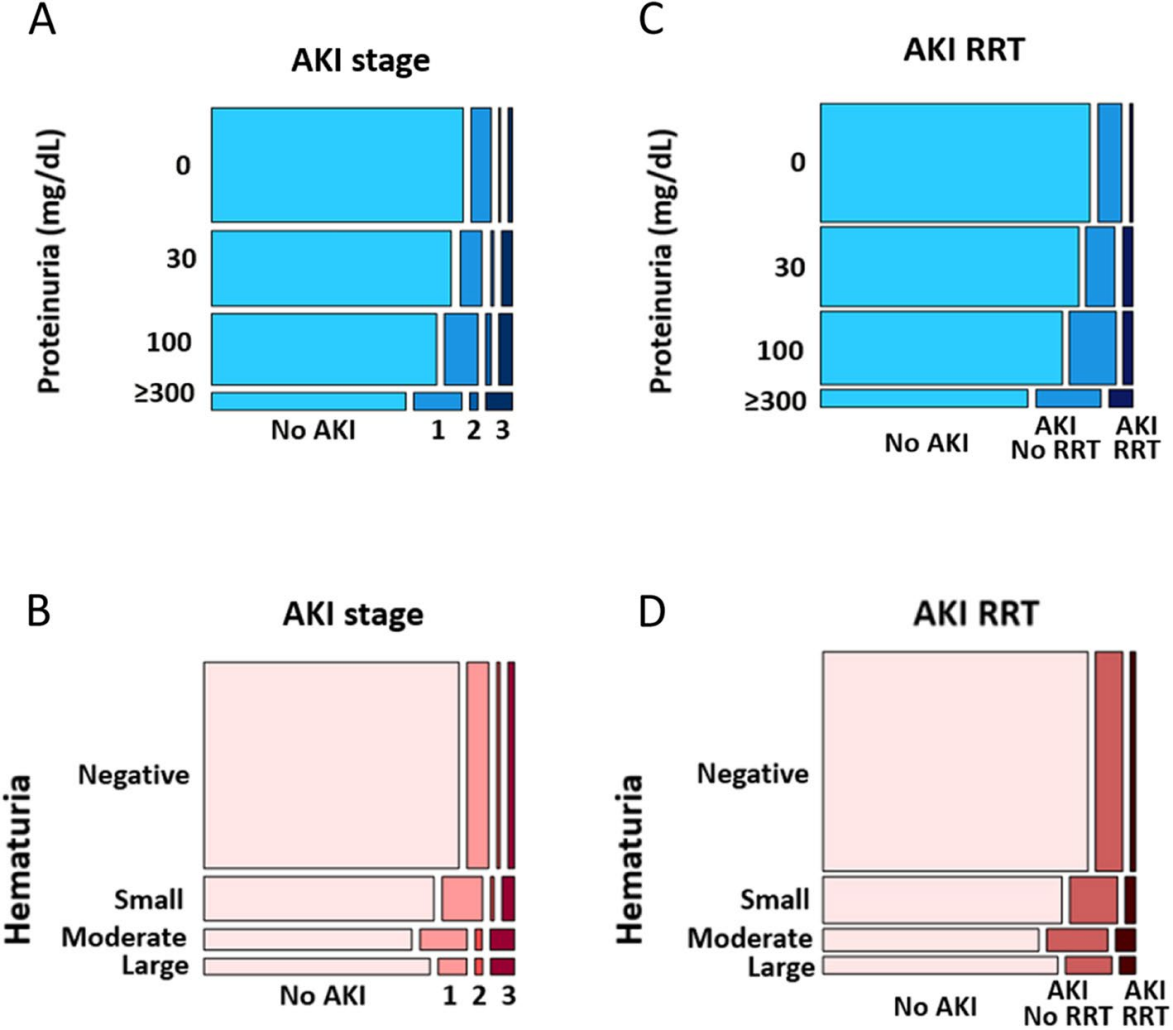
Severity of urinary abnormalities by AKI status, stage, and subsequent need for RRT. Higher AKI stage was associated with an increasing degree of **A** proteinuria, *P*-value < 0.001 and **B** hematuria, *P*-value < 0.001; while development of AKI without RRT (AKI no RRT) and progression to requiring RRT (AKI RRT) were associated with an increasing degree of **C** proteinuria and **D** hematuria. Linear-by-linear association tests for ordered multilevel categorical variables were used. There was a monotonic increase in the percentage of AKI stages and AKI not requiring or requiring RRT as proteinuria and hematuria severity increased. *P* < 0.001, proteinuria vs. AKI stage (**A**), hematuria vs. AKI stage (**B**), proteinuria vs. AKI RRT (**C**), hematuria vs. AKI RRT (**D**). Abbreviations: AKI stage, KDIGO stages of Acute Kidney Injury (AKI) 1, 2, and 3, with stage 3 being most severe; AKI no RRT, development of AKI during admission but not requiring renal replacement therapy (RRT); AKI RRT, AKI requiring RRT; Hematuria, degree of blood present on dipstick urinalysis, categorized as negative, small, moderate, and large; Proteinuria, degree of proteinuria present on dipstick urinalysis, categorized as 0, 30, 100, and ≥300

### Outcome Measures

The pre-specified primary outcome was development of AKI. The secondary outcome was AKI requiring RRT. We used an algorithm based on the Kidney Disease Improving Global Outcomes (KDIGO) guidelines to determine presence and stage of AKI (Additional Fig. 2). We did not use urine output criteria given concerns for accurate documentation in the EHR. Patients, who met criteria for AKI, were excluded if the AKI was present on admission (initial inpatient creatinine value greater than 0.3 mg/dl or 1.5 times the baseline creatinine).

**Fig. 2:**
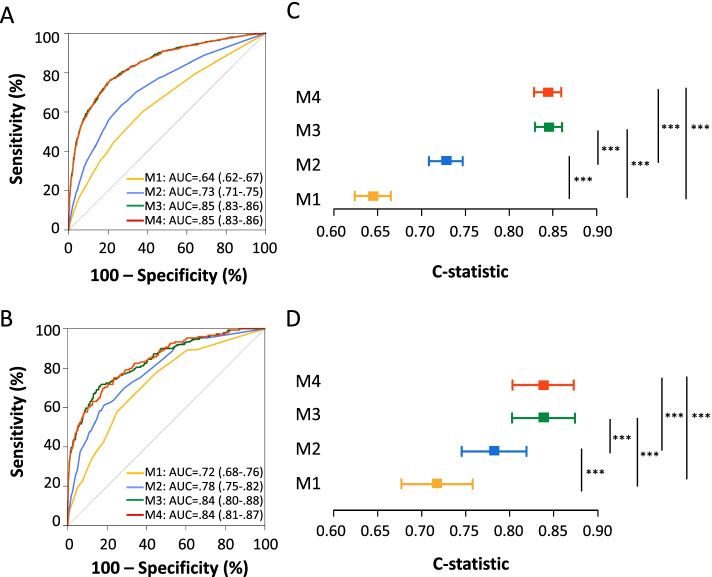
Receiver operating characteristic curves (ROC) for **A** AKI and **B** RRT predictive models and C-statistics for **C** AKI models and **D** RRT models. *** represents *P* < 0.001. *P* values are for Delong’s tests comparing nested models, and are adjusted by the Holm’s method. Model 1 (M1)—Level of hematuria and proteinuria on dipstick proteinuria. Model 2 (M2)—Model 1 + CKD presence. Model 3 (M3)—Model 1 + baseline creatinine. Model 4 (M4) - Model 1 + CKD presence + baseline creatinine

### Statistical Analysis

We separated patients into *AKI categories* – no AKI, AKI not requiring RRT (AKI no RRT), and AKI requiring RRT (AKI RRT) – and *AKI stages* (no AKI, AKI stage 1, stage 2, and stage 3). We reported continuous variables as median and interquartile ranges (IQR) and presented categorical variables as proportions. To compare across AKI categories, we used the Cochran-Armitage Trend test for binary categorical variables. We performed the linear-by-linear association test for ordered multilevel categorical variables and we applied the Jonckheere-Terpstra test for continuous variables. Pairwise comparisons among no AKI, AKI no RRT, and AKI RRT were performed when the overall test was significant, and the adjustment to *P* values was based on Holm’s method.

To build prognostic models for AKI and RRT, we evaluated the patient group without AKI on admission. We build nested logistic models on top of a base *Model 1* which included dipstick proteinuria and hematuria. *Model 2* added CKD to *Model 1*, *Model 3* added baseline creatinine to *Model 1*, and *Model 4* added both CKD and baseline creatinine to *Model 1*. We compared nested models using likelihood ratio tests. We evaluated areas under the receiver-operation characteristic (ROC) curve (AUC) with DeLong’s tests. We assessed models for multi-collinearity using variance inflation factors (VIF), where a VIF greater than ten suggests evidence of multicollinearity. Model evaluation indices contained sensitivity, specificity, positive predictive value (PPV), negative predictive value (NPV), positive likelihood ratio (PLR), and negative likelihood ratio (NLR), shown as an average (± standard error of the mean, SEM) across tenfold stratified cross validation (CV). We used the Youden’s index from ROC analysis to identify the optimal predicted probability cutoff. We derived mathematic equations to calculate the log odds (logit) for AKI and RRT and converted them into the predicted probability of AKI and RRT.

We performed all statistical analyses using R software, version R-4.1.0 (R Foundation for Statistical Computing). A 2-sided *P* value of less than 0.05 was considered statistically different.

## Results

### Baseline characteristics

We included 5,980 individuals, of whom 829 (13.9%) developed an AKI during admission. Of those, 149 (18.0%) received RRT. There were 680 (11.4%) individuals, who met criteria for AKI but did not receive RRT, 149 (2.5%), who met criteria for AKI and did receive RRT, and 5,151 (86.1%), who did not meet criteria for AKI. The demographics, medical comorbidities, and laboratory variables can be reviewed in Table 1 based on AKI and RRT status. In the entire cohort 2,900 (48.5%) were male, 882 (14.8%) were Black, and 2,135 (35.7%) were Hispanic. Patients with AKI were older, more likely to be male, have underlying hypertension, diabetes mellitus, CKD, and congestive heart failure (CHF). Patients with AKI had a statistically significantly higher baseline creatinine than those without AKI (*P* < 0.001). Of patients with AKI, those who received RRT were more likely to be male and have underlying CKD than those with AKI who did not receive RRT. Those with AKI not requiring RRT were more likely to have coronary artery disease (CAD) and be prescribed an angiotensin converting enzyme inhibitor (ACEI) or angiotensin receptor blocker (ARB) at baseline than those who did receive RRT.

**Table 1:** Baseline Characteristics and laboratory values based on AKI and RRT presence

**Characteristic**	**No AKI** **no. (%)** ***N* = 5151**	**AKI no RRT** **no. (%)** ***N* = 680**	**AKI RRT** **no. (%)** ***N* = 149**	*P* value^*^
Age, years, median [IQR]	60.0 [47.0, 73.0]^1c, 2a^	69.0 [56.0, 79.0]^3a^	64.0 [54.0, 73.0]	< 0.001
Male sex	2407 (46.7)^1c, 2c^	391 (57.5)^3a^	102 (68.5)	< 0.001
Hispanic ethnicity	1867 (37.3)	202 (31.1)	66 (45.2)	0.430
Black Race	750 (15.3)	105 (16.4)	27 (19.1)	0.174
Smoker (smoked at any time)	1648 (32.3)^1c^	267 (40.1)	50 (34.0)	0.002
Hypertension	2778 (53.9)^1c, 2c^	485 (71.3)	107 (71.8)	< 0.001
Diabetes Mellitus	1643 (31.9)^1c, 2b^	315 (46.3)	67 (45.0)	< 0.001
CKD	845 (16.4)^1c, 2c^	315 (46.3)^3b^	88 (59.1)	< 0.001
CAD	521 (10.1)^1c^	151 (22.2)^3b^	15 (10.1)	< 0.001
CHF	404 (7.8)^1c, 2a^	126 (18.5)	21 (14.1)	< 0.001
Cirrhosis	87 (1.7)	16 (2.4)	4 (2.7)	0.142
COPD	487 (9.5)^1c^	101 (14.9)	15 (10.1)	0.001
Statin (on presentation)	1892 (36.7)^1c^	340 (50.0)	66 (44.3)	< 0.001
ACEI/ARB	1803 (35.0)^1c^	318 (46.8)^3b^	50 (33.6)	< 0.001
**Laboratory Values**				
Baseline serum creatinine, mg/dL, median [IQR]	0.8 [0.7, 1.0]^1c, 2c^	1.4 [1.0, 1.8]	1.8 [1.0, 4.0]	< 0.001
Proteinuria				< 0.001
Negative	2237 (43.4)^1c, 2c^	199 (29.3)^3b^	23 (15.4)	
30 mg/dL	1413 (27.4)	161 (23.7)	51 (34.2)	
100 mg/dL	1236 (24.0)	237 (34.9)	45 (30.2)	
≥ 300 mg/dL	265 (5.1)	83 (12.2)	30 (20.1)	
Hematuria				< 0.001
Negative	3828 (74.3)^1c, 2c^	390 (57.4)^3b^	71 (47.7)	
Small	736 (14.3)	147 (21.6)	31 (20.8)	
Moderate	318 (6.2)	90 (13.2)	29 (19.5)	
Large	269 (5.2)	53 (7.8)	18 (12.1)	

*AKI,* acute kidney injury, *IQR* interquartile range, *CKD* chronic kidney disease, *CAD* coronary artery disease, *CHF* congestive heart failure, *COPD* chronic obstructive pulmonary disease, *ACEI/ARB* angiotensin-converting enzyme inhibitors/angiotensin II receptor blockers

Comparing no AKI, AKI no RRT, and AKI RRT by Cochran-Armitage Trend test for binary categorical variables, linear-by-linear association test for ordered multilevel categorical variables (proteinuria and hematuria), and Jonckheere-Terpstra test for continuous variables

Pairwise comparisons among no AKI, AKI not received RRT, and AKI received RRT were performed if overall test was significant *P* values were adjusted with Holm’s method

^1a^*P* < 0.05, ^1b^*P* < 0.01, ^1c^*P* < 0.001. Pairwise comparison between no AKI and AKI not received RRT

^2a^*P* < 0.05, ^2b^*P* < 0.01, ^2c^*P* < 0.001. Pairwise comparison between no AKI and AKI received RRT

^3a^*P* < 0.05, ^3b^*P* < 0.01, ^3c^*P* < 0.001. Pairwise comparison between AKI not received RRT and AKI received RRT

### Urinary Abnormalities

Percentages for each cutoff for proteinuria and hematuria based on AKI and RRT status can also be seen in Table 1. These are illustrated graphically based on AKI stages in Figs. 1A and 1B and no AKI, AKI no RRT, and AKI RRT categories in Figs. 1C and 1D**.** There was a statistically significant trend for a higher percentage of proteinuria and hematuria as AKI severity increased (*P* < 0.001 across advancing AKI stages; linear-by-linear association test *P* < 0.001 across presence of AKI not requiring or requiring RRT). Further, by pairwise comparison, a significantly higher percentage of participants with proteinuria and hematuria was found among those with AKI not requiring RRT *vs.* patients without AKI (adjusted *P* < 0.001 for both proteinuria and hematuria) and patients with AKI requiring RRT *vs.* those with AKI not requiring RRT (adjusted *P* = 0.007 for proteinuria, adjusted *P* = 0.006 for hematuria, Cochran-Armitage test for trend.)

### Predictors of AKI during Hospitalization

All levels of proteinuria and hematuria were associated with a statistically significant increased risk of AKI (Table 2). Baseline serum creatinine was associated with an increase in odds of AKI, with an odds ratio of 9.90 (95% CI, 8.22, 11.92) per 1 mg/dL of creatinine. The presence of CKD had an odds ratio of 4.82 (95% CI, 4.13, 5.63) for developing AKI. Figs. 2A and 2B, respectively, display the ROC curves for nested AKI and AKI RRT prediction models. Figs. 2C and 2D illustrate the corresponding Forest plots for AUC comparisons. *Model 1* contained level of proteinuria and level of hematuria, with an AUC of 0.64 (95% CI 0.62, 0.67). *Model 2* added the presence of CKD to the variables in *Model 1* with an AUC improvement to 0.73 (95% CI 0.71, 0.75), adjusted *P* for Delong’s test < 0.001 *vs. Model 1*. *Model 3* added baseline creatinine to variables in *Model 1* with an improvement in AUC to 0.85 (95% CI 0.83, 0.86), adjusted *P* for Delong’s test < 0.001 *vs. Model 1*. *Model 4* contained level of proteinuria, level of hematuria, presence of CKD, and baseline creatinine with an AUC of 0.85 (95% CI 0.83, 0.86). *Model 4* showed improvement in AUC when compared to *Model 1,* adjusted *P* < 0.001, and also to *Model 2, P* < 0.001. However, there was no significant improvement in model discrimination when we added CKD to baseline serum creatinine (*Model 4* vs. *Model 3*) (Fig. 2B). No severe multicollinearity was seen by VIF analysis.

**Table 2:** Univariable and multivariable predictive models for AKI

Variable	Univariable Model OR (95% CI)	*p*	Model 1 OR (95% CI)	Model 2 OR (95% CI)	Model 3 OR (95% CI)	Model 4 OR (95% CI)
Proteinuria						
Negative	Reference					
30	1.51 (1.24, 1.85)	< 0.001	1.42 (1.16, 1.74)	1.37 (1.11, 1.68)	1.28 (1.02, 1.59)	1.28 (1.02, 1.59)
100	2.30 (1.90, 2.78)	< 0.001	1.97 (1.62, 2.39)	1.78 (1.45, 2.17)	1.29 (1.03, 1.62)	1.29 (1.03, 1.62)
≥ 300	4.30 (3.32, 5.57)	< 0.001	3.35 (2.56, 4.39)	2.60 (1.96, 3.44)	1.52 (1.09, 2.13)	1.49 (1.06, 2.08)
Hematuria						
Negative	Reference					
Small	2.01 (1.66, 2.43)	< 0.001	1.69 (1.39, 2.05)	1.58 (1.29, 1.94)	1.41 (1.12, 1.77)	1.40 (1.11, 1.77)
Moderate	3.11 (2.46, 3.92)	< 0.001	2.51 (1.98, 3.19)	2.34 (1.83, 3.01)	2.05 (1.54, 2.72)	2.05 (1.55, 2.72)
Large	2.19 (1.66, 2.90)	< 0.001	1.69 (1.27, 2.25)	1.69 (1.25, 2.29)	1.77 (1.25, 2.50)	1.76 (1.25, 2.50)
Baseline Creatinine	9.90 (8.20, 11.92)	< 0.001			8.98 (7.44, 10.82)	7.59 (6.19, 9.31)
CKD	4.82 (4.13, 5.63)	< 0.001		4.29 (3.66, 5.03)		1.44 (1.18, 1.77)

*AKI* acute kidney injury, *OR* odds ratio, *CI* confidence interval, *CKD* chronic kidney disease. Model 1 includes level of proteinuria and hematuria; Model 2 includes Model 1 plus presence of CKD; Model 3 includes Model 1 plus baseline creatinine; Model 4 includes Model 1 plus presence of CKD and baseline creatinine

We derived formulas for prediction of AKI using these models. Table 3 shows formulas for computing the logit for AKI for each model, which can then be used to calculate the predicted probability of AKI. We describe performance metrics, such as sensitivity, specificity, positive and negative predictive values, and positive and negative likelihood ratios, for AKI predictive models in Additional Table 1.

**Table 3:** Formulas for AKI prediction

Model	Logit AKI =
Model 1	-2.4452 + 0.3522(Proteinuria_1^a^) + 0.676(Proteinuria_2^a^) + 1.2096(Proteinuria_3^a^) + 0.5222(Hematuria_1^a^) + 0.9211(Hematuria_2^a^) + 0.525(Hematuria_3^a^)
Model 2	-2.8122 + 0.3126(Proteinuria_1^a^) + 0.5726(Proteinuria_2^a^) + 0.9538(Proteinuria_3^a^) + 0.4577(Hematuria_1^a^) + 0.8515(Hematuria_2^a^) + 0.5267(Hematuria_3^a^) + 1.4569(CKD^a^)
Model 3	-4.647 + 0.2436(Proteinuria_1^a^) + 0.2548(Proteinuria_2^a^) + 0.4193(Proteinuria_3^a^) + 0.3407(Hematuria_1^a^) + 0.7163(Hematuria_2^a^) + 0.5716(Hematuria_3^a^) + 2.1944(Baseline Creatinine)
Model 4	4.5705 + 0.2431(Proteinuria_1^a^) + 0.2557(Proteinuria_2^a^) + 0.3953(Proteinuria_3^a^) + 0.3379(Hematuria_1^a^) + 0.7184(Hematuria_2^a^) + 0.567(Hematuria_3^a^) + 2.027(CKD^a^) + 0.3668(Baseline Creatinine)

*AKI* acute kidney injury, *CKD* chronic kidney disease. Model 1 includes level of proteinuria and hematuria; Model 2 includes Model 1 plus presence of CKD; Model 3 includes Model 1 plus baseline creatinine; Model 4 includes Model 1 plus presence of CKD and baseline creatinine

The logit AKI can be transformed into the predicted probability of AKI with the following formula: Predicted AKI = 1/(1 + e^(−1*Logit AKI)^)

^a^For patients that have CKD, insert 1; for patients having 30 mg/dL protein by dipstick test insert 1 for Proteinuria_1; for patients having 100 mg/dL protein by dipstick test insert 1 for Proteinuria_2; for patients having ≥ 300 mg/dL by dipstick test insert 1 for Proteinuria_3; for patients having small by dipstick test insert 1 for Hematuria _1; for patients having moderate by dipstick test insert 1 for Hematuria _2; for patients having large by dipstick test insert 1 for Hematuria _3, otherwise insert 0

### Predictors of RRT during Hospitalization

All levels of proteinuria and hematuria were associated with a statistically significant increase in risk for AKI requiring RRT (Table 4). Baseline serum creatinine was associated with an increase in odds of RRT, with an odds ratio of 2.22 (95% CI 1.99, 2.48) per 1 mg/dL creatinine. Presence of CKD diagnosis had an odds ratio of 5.81 (95% CI 4.16, 8.10) for receiving RRT. ROC curves for nested RRT models can be reviewed in Fig. 2B**.** *Model 1* for predicting RRT contained level of proteinuria and level of hematuria with an AUC of 0.72 (95% CI 0.68, 0.76). *Model 2* added presence of CKD to variables in *Model 1* and AUC improved to 0.78 (95% CI 0.75, 0.82), adjusted *P* < 0.001. *Model 3* added baseline creatinine to variables in *Model 1* with an improvement in AUC of 0.84 (95% CI 0.80, 0.87), adjusted *P* < 0.001. *Model 4* contained level of proteinuria, level of hematuria, presence of CKD, and baseline creatinine with an AUC of 0.84 (95% CI 0.81, 0.87), adjusted *P* < 0.001 *vs. Model 1,* adjusted *P* < 0.001 *vs. Model 2*. However the improvement was not significant when *Model 4* was compared to *Model 3* (Fig. 2D)**.** No severe multicollinearity was seen by VIF analysis. Table 5 shows formulas for computing the logit for RRT for each model which can then be used to calculate the predicted probability of RRT. Performance metrics for RRT predictive models can be seen in Additional Table 1.

**Table 4:** Univariable and predictive models for AKI RRT

Variable	Univariable Model OR (95% CI)	*p*	Model 1 OR (95% CI)	Model 2 OR (95% CI)	Model 3 OR (95% CI)	Model 4 OR (95% CI)
Proteinuria						
Negative	Reference					
30	3.43 (2.09,5.64)	< 0.001	3.13 (1.90, 5.15)	3.01 (1.82, 4.97)	2.92 (1.76, 4.84)	2.89 (1.74, 4.79)
100	3.24 (1.95, 5.37)	< 0.001	2.58 (1.54, 4.33)	2.22 (1.31, 3.74)	1.51 (0.86, 2.65)	1.44 (0.82, 2.53)
≥ 300	9.13 (5.24, 15.90)	< 0.001	6.51 (3.66, 11.56)	4.61 (2.56, 8.28)	2.24 (1.11, 4.50)	2.06 (1.03, 4.10)
Hematuria						
Negative	Reference					
Small	2.09 (1.36, 3.20)	< 0.001	1.66 (1.07, 2.58)	1.50 (0.96, 2.34)	1.18 (0.71, 1.97)	1.16 (0.70, 1.92)
Moderate	4.22 (2.71, 6.58)	< 0.001	3.16 (2.00, 4.99)	2.83 (1.77, 4.51)	2.60 (1.55, 4.34)	2.48 (1.49, 4.13)
Large	3.32 (1.96, 5.64)	< 0.001	2.40 (1.39, 4.13)	2.35 (1.35, 4.09)	2.06 (1.11, 3.84)	2.05 (1.10, 3.82)
Baseline Creatinine	2.22 (1.99, 2.48)	< 0.001			2.14 (1.89, 2.42)	1.89 (1.67, 2.15)
CKD	5.81 (4.17, 8.10)	< 0.001		4.82 (3.43, 6.78)		2.44 (1.64, 3.64)

*RRT* renal replacement therapy, *OR* odds ratio, *CI* confidence interval, *CKD* chronic kidney disease. Model 1 includes level of proteinuria and hematuria; Model 2 includes Model 1 plus presence of CKD; Model 3 includes Model 1 plus baseline creatinine; Model 4 includes Model 1 plus presence of CKD and baseline creatinine

**Table 5:** Formulas for RRT prediction

Model	Logit RRT =
Model 1	- 4.8378 + 1.1405(Proteinuria_1^a^) + 0.9478(Proteinuria_2^a^) + 1.8725(Proteinuria_3^a^) + 0.5065(Hematuria_1^a^) + 1.149(Hematuria_2^a^) + 0.8743(Hematuria_3^a^)
Model 2	-5.3054 + 1.1009(Proteinuria_1^a^) + 0.7955(Proteinuria_2^a^) + 1.5277(Proteinuria_3^a^) + 0.4078(Hematuria_1^a^) + 1.0389(Hematuria_2^a^) + 0.8529(Hematuria_3^a^) + 1.5733(CKD^a^)
Model 3	-5.5353 + 1.0716(Proteinuria_1^a^) + 0.4113(Proteinuria_2^a^) + 0.8066(Proteinuria_3^a^) + 0.1691(Hematuria_1^a^) + 0.9542(Hematuria_2^a^) + 0.7245(Hematuria_3^a^) + 0.7596(Baseline Creatinine)
Model 4	-5.6733 + 1.0601(Proteinuria_1^a^) + 0.3666(Proteinuria_2^a^) + 0.7205(Proteinuria_3^a^) + 0.1472(Hematuria_1^a^) + 0.9078(Hematuria_2^a^) + 0.7181(Hematuria_3^a^) + 0.6386(CKD*) + 0.8924(Baseline Creatinine)

*RRT* renal replacement therapy, *CKD* chronic kidney disease. Model 1 includes level of proteinuria and hematuria; Model 2 includes Model 1 plus presence of CKD; Model 3 includes Model 1 plus baseline creatinine; Model 4 includes Model 1 plus presence of CKD and baseline creatinine. The logit RRT can be transformed into the predicted probability of RRT with the following formula: Predicted RRT = 1/(1 + e^(−1*Logit RRT)^)

^a^For patients that have CKD, insert 1; for patients having 30 mg/dL protein by dipstick test insert 1 for Proteinuria_1; for patients having 100 mg/dL protein by dipstick test insert 1 for Proteinuria_2; for patients having ≥ 300 mg/dL by dipstick test insert 1 for Proteinuria_3; for patients having small by dipstick test insert 1 for Hematuria _1; for patients having moderate by dipstick test insert 1 for Hematuria _2; for patients having large by dipstick test insert 1 for Hematuria _3, otherwise insert 0

## Discussion

As a frequent non-pulmonary manifestation of COVID-19, AKI is an important factor in the pathophysiology of SARS-CoV-2 infection, adverse outcome prediction, and issues encompassing RRT resource utilization in the setting of the pandemic. In this large, multiethnic cohort of 5,980 individuals hospitalized with COVID-19, who had a urinalysis obtained and did not present with AKI at admission, we showed that 14% developed AKI and of those, 18% received RRT. The rate of AKI we found is less than the previously reported rates of AKI as we excluded AKI that was present on admission to develop our predictive models. We demonstrated that escalating severity of dipstick proteinuria or hematuria were associated with the development of AKI and need for RRT in a dose–response fashion. Importantly, we derived formulas containing dipstick urinalysis and serum creatinine, which are frequently obtained in hospitalized patients, to help clinicians quickly determine risk for poor kidney outcomes and allow early decisions to mitigate these risks.

Proteinuria has been previously shown to be associated with incident AKI in some severe non-COVID-19 conditions requiring hospitalizations, such as critical illness [24], cirrhosis [25], and burns [26]. One study reported associations of proteinuria on pre-operative urinalysis with an increased risk of AKI and need for post-operative RRT in patients undergoing coronary artery bypass graft surgery [27]. Another showed that pre-operative urine albumin-to-creatinine ratio was associated with need for RRT post-operatively in patients undergoing cardiac surgery and improved clinical models over dipstick proteinuria for predicting AKI [28]. Only one report evaluated the association between hematuria and risk for AKI, showing an association between dipstick hematuria greater than 1 + and AKI in patients with critical illness [24]. However, findings in these patient populations may not be generalizable to patients hospitalized with COVID-19. Recently, Patel et al*.* did show that hematuria and proteinuria present on dipstick urinalysis were associated with AKI in patients hospitalized with COVID-19 [17]. We validate these findings in a larger, ethnically diverse cohort of patients from a greater number of hospitals and show that presence of protein and blood on a simple and widely clinically available test, the dipstick urinalysis, was associated with AKI development during hospitalization in patients infected with COVID-19, as well as with the need for RRT. We further extend these findings by developing a predictive tool for both AKI and AKI requiring RRT which utilizes the dipstick urinalysis findings and can easily be used by clinicians to risk stratify patients. By excluding those patients with AKI present on admission, we enhance the predictive value of these tools. 

The unique kidney pathology associated with COVID-19 highlights the importance of developing AKI risk prediction models specific to this population. Acute tubular necrosis may be a common etiology of AKI [29], as it is in other hospital settings; however, data also support virus-mediated injury involving cytokine storm, thrombotic microangiopathy, and glomerulonephritis as possible causes of kidney injury in COVID-19 [5]. Our findings that proteinuria and hematuria on dipstick urinalysis are associated with increased risk for AKI may suggest presence of subclinical kidney pathology *prior* to clinically apparent AKI. In other words, if SARS-CoV-2 infection directly affects the tubular epithelium and/or the podocyte, the proteinuria and hematuria seen on urinalysis may represent an earlier phase of AKI that precedes the rise in serum creatinine. Alternatively, the findings could be consistent with the heuristic in nephrology that those with preexisting kidney disease are more at risk for developing AKI in the setting of other acute illnesses supported by the fact that adding CKD presence and baseline creatinine to the model improved the prognostic capability of the model. Given that those who developed AKI carried much higher rates of documented CKD, diabetes mellitus, and hypertension, these findings may represent underlying chronic disease rather than acute changes in dipstick urinalysis results. However, given that the risk of AKI was also increased in models that only included proteinuria and hematuria, it is possible to infer that dipstick proteinuria and hematuria are indicative of an active urinary sediment and additional kidney pathology beyond the presence of prior CKD. Overall, our findings support that AKI in the setting of COVID-19 is likely multifactorial and may not be limited to acute tubular necrosis related to severe acute illness.

In addition to extending the associations of proteinuria and hematuria on urinalysis with the development of AKI to individuals hospitalized with COVID-19, we derived formulas to predict the risk of AKI and need for RRT using the presence and degree of proteinuria and hematuria. We showed that adding either baseline creatinine or CKD presence can be used to help improve the prediction of both AKI and RRT, depending on the information available to the clinician. A potential explanation as to why there was no significant improvement in model discrimination when both serum creatinine and CKD are included in the model could be that both variables may be measuring a similar construct. This information is valuable for clinicians so that either presence of CKD or baseline creatinine can be used to predict AKI if only one of these variables is known in a given patient. For example, using our formulas, if a patient has 100 mg/dL of proteinuria and “moderate” hematuria on initial urinalysis plus CKD, it can be determined that the patient has a 52% chance of developing AKI and a 13% chance of receiving RRT during hospitalization with COVID-19.

These risk prediction formulas will be helpful to providers as well as institutions in management of illness, estimating resource allocation, and setting family expectations. Additionally, predicting risk of RRT would help allocate nursing, supplies, and other resources to be better prepared. Most importantly, studies have shown a higher mortality for patients with AKI [11, 30]. The risk prediction can be assessed on admission and used in conjunction with other clinical parameters to better prepare patients and families about potential outcomes.

Our study does have some important limitations requiring discussion. First, we were unable to determine if the initial urinalysis samples were obtained using urinary catheters, which may lead to positive dipstick hematuria from urethral trauma in the absence of glomerular bleeding or other kidney pathology related to COVID-19. However, given that we used the first available urinalysis test obtained upon hospitalization, which was within the first 24 h of admission for 87% of the participants and within the first 48 h for 93% of participants, we limited this possibility. Second, although a dipstick urinalysis is a relatively inexpensive test usually obtained upon hospitalization, we recognize that some selection bias may have been imparted by excluding about a quarter of the patients hospitalized with COVID-19 who did not have an available urinalysis. Third, data were obtained from the EHR, largely using billing codes, which may affect the accuracy of information about comorbidities such as CKD. This likely resulted in lower positive predictive values for the models, but importantly increased the negative predictive values and the specificity of the derived formulas. Finally, we acknowledge that there is currently no standardized definition for estimating baseline creatinine, and that various methods used may lead to potential bias [31].

## Conclusions

In a large, multiethnic, contemporary cohort of patients hospitalized with COVID-19, we showed that proteinuria and hematuria on dipstick urinalysis were associated with increased risk for developing AKI and for receiving RRT. Prediction models including presence and degree of proteinuria and hematuria in combination with CKD status or baseline creatinine accurately predicted both AKI and RRT. Our models include variables that are routinely clinically available and can easily be utilized by clinicians to prognosticate AKI in hospitalized patients with COVID-19.

## Supplementary Information

**Additional file 1.** 

## Data Availability

The datasets used and analyzed during this study are available from the corresponding author on reasonable request. At this time we are not able to share the data publicly because our research group is working on several other analyses from the same data that will be subsequently submitted for publication.

## Abbreviations

ACEI: Angiotensin converting enzyme inhibitor
AKI: Acute kidney injury
ARB: Angiotensin receptor blocker
AUC: Area under the curve
CAD: Coronary artery disease
CHF: Congestive heart failure
CI: Confidence interval
CKD: Chronic kidney disease
COVID-19: Coronavirus disease 2019
CV: Cross validation
EHR: Electronic health record
ESKD: End stage kidney disease
KDIGO: Kidney Disease Improving Global Outcomes
NPV: Negative predictive value
NLR: Negative likelihood ratio
PLR: Positive likelihood ratio
PPV: Positive predictive value
ROC: Receiver-operating characteristic
RRT: Renal replacement therapy
SARS-CoV2: Severe acute respiratory syndrome-coronavirus 2
SEM: Standard error of the mean
UPCR: Urine protein-to-creatinine ratio
UTSW: University of Texas Southwestern
VIF: Variance inflation factor
