# Supplementary material for: Using dipstick urinalysis to predict development of acute kidney injury in patients with COVID-19
Source: BMC Nephrol. 2022 Feb 1;23:50. doi: 10.1186/s12882-022-02677-y (PMC8805668; doi:10.1186/s12882-022-02677-y)
Supplement: Supplementary file 1 — Additional file 1. [file 12882_2022_2677_MOESM1_ESM.pdf]

Supplemental Material Table of Contents

Supplemental Figure 1. Algorithm to determine baseline creatinine.

Supplemental Figure 2. Algorithm to determine presence of AKI as well as stage of AKI and present on admission (POA) AKI.

Supplemental Table 1. Performance of predictive models for AKI and RRT.

Supplemental Figure 1. Algorithm for determination of baseline creatinine (Cr_0). Cr refers to creatinine value. ESRD, end stage renal disease. Cr, creatinine. *Renal replacement therapy (RRT) includes continuous renal replacement therapy (CRRT), hemodialysis (HD), or peritoneal dialysis (PD).

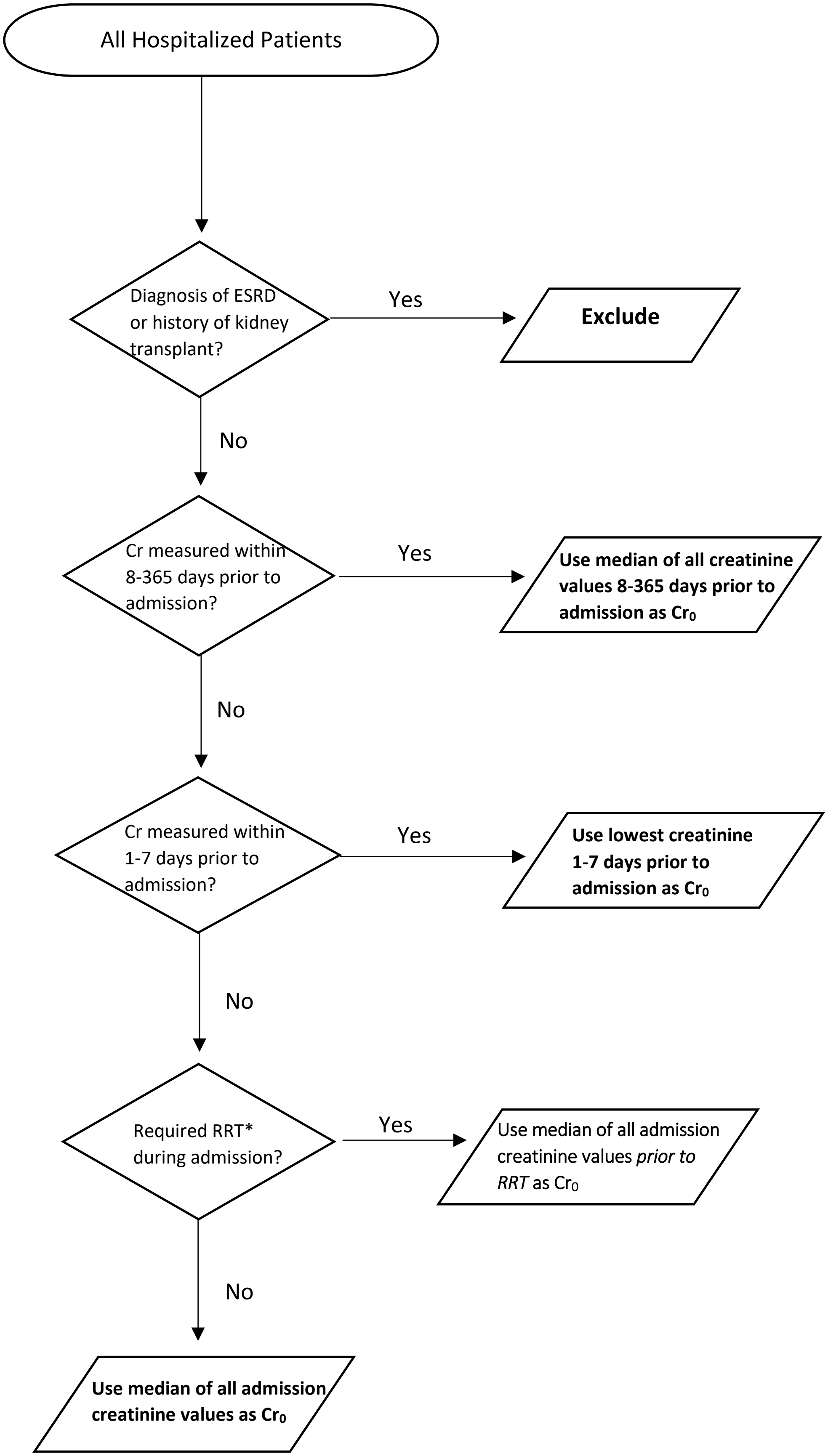

Supplemental Figure 2. Algorithm to determine presence and stage of AKI as well as presence of AKI on admission (POA AKI). *Renal replacement therapy (RRT) includes continuous renal replacement therapy (CRRT), hemodialysis (HD), or peritoneal dialysis (PD). ESRD, end stage renal disease. Cr, creatinine. $Cr_{0,}$ baseline creatinine.

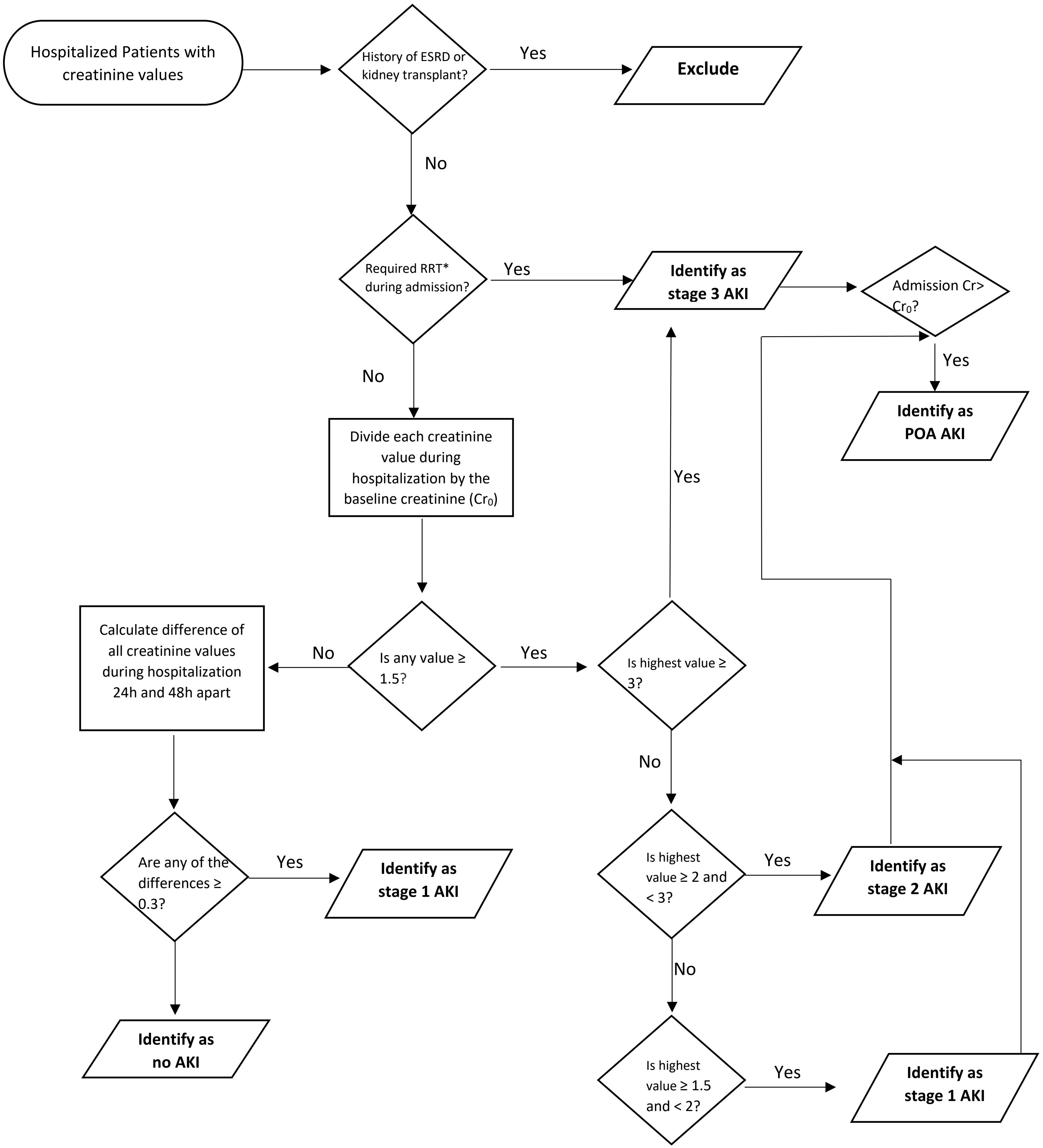

Supplemental Table 1. Performance of predictive models for AKI and RRT.

**AKI**				
**Metric, mean ±SEM**	**Model 1**	**Model 2**	**Model 3**	**Model 4**
AUC	0.642±0.014	0.726±0.008	0.844±0.008	0.842±0.007
Sensitivity	0.563±0.042	0.659±0.031	0.754±0.027	0.749±0.024
Specificity	0.684±0.032	0.728±0.025	0.812±0.017	0.812±0.016
PPV	0.227±0.01	0.286±0.011	0.404±0.019	0.4±0.016
NPV	0.908±0.005	0.931±0.004	0.954±0.004	0.953±0.004
PLR	1.842±0.104	2.512±0.13	4.303±0.348	4.213±0.293
NLR	0.63±0.038	0.462±0.031	0.299±0.028	0.306±0.025
**RRT**				
**Metric, mean ±SEM**	**Model 1**	**Model 2**	**Model 3**	**Model 4**
AUC	0.711±0.019	0.776±0.015	0.832±0.02	0.83±0.02
Sensitivity	0.771±0.047	0.732±0.039	0.752±0.037	0.751±0.046
Specificity	0.609±0.046	0.747±0.054	0.839±0.042	0.819±0.032
PPV	0.053±0.005	0.086±0.01	0.151±0.027	0.117±0.016
NPV	0.991±0.001	0.991±0.001	0.993±0.001	0.993±0.001
PLR	2.186±0.216	3.715±0.473	7.446±1.67	5.299±0.833
NLR	0.352±0.057	0.342±0.035	0.29±0.035	0.293±0.05

AKI, acute kidney injury. RRT, renal replacement therapy.

Model 1 includes level of proteinuria and hematuria, Model 2 includes Model 1 plus presence of CKD, Model 3 includes Model 1 plus baseline creatinine, and Model 4 includes Model 1 plus presence of CKD and baseline creatinine.

SEM, standard error of the mean. AUC, area under the curve. PPV, positive predictive value. NPV, negative predictive value. PLR, positive likelihood ratio. NLR, negative likelihood ratio.
